# Serum Interleukin-18 and Its Gene Haplotypes Profile as Predictors in Patients with Diabetic Nephropathy

**DOI:** 10.3889/oamjms.2016.074

**Published:** 2016-07-21

**Authors:** Ahmed I. Abd Elneam, Nahla M. Mansour, Nayel A. Zaki, Mohamed A. Taher

**Affiliations:** 1 *Molecular Genetics and Enzymology Dept., Human Genetics Division, National Research Centre, 33 El Bohouth St. (former El Tahrir St.), Dokki 12622, Cairo, Egypt (Affiliation ID 60014618)*; 2 *Gut Microbiology and Immunology Group, Chemistry of Natural and Microbial Products Department, Pharmaceutical Industries Research Division, National Research Centre, 33 El Bohouth St. (former El Tahrir St.), Dokki 12622, Cairo, Egypt*; 3 *Internal Medicine Department, Faculty of Medicine, Sohag University, Sohag, Egypt*; 4 *Medical Biochemistry Department, Sohag Faculty of Medicine, Sohag University, Egypt*

**Keywords:** Diabetic Nephropathy, IL-18- polymorphisms, PCR, RFLP, haplotype

## Abstract

**BACKGROUND:**

Diabetic nephropathy (DN) is known as an acute microvascular complexity as a subsequence progression in diabetes mellitus type 1 and 2. Many evidence pointed that the proinflammatory cytokine Interleukin (IL)-18 might be involved in the pathogenesis of DN.

**AIM:**

The current study aimed to evaluate the association of serum IL-18 and its promoter gene polymorphisms with diabetic nephropathy.

**METHODS:**

This study included 62 diabetic nephropathy patients (DN group) compared to 52 diabetes mellitus patients (DM group). The two groups were subjected to anthropometry assessment, molecular studies including SNP genotyping by RFLP and finally statistical analysis.

**RESULTS:**

The assessment of the serum IL-18 level and the frequencies of its allele and haplotype: *-137G/C*, *-607C/A* and *-656G/T* among the DN and DM subjects revealed that -137G allele has significant variation between DN and DM subjects (about 80.8%, P = 0.05) but, no significant variation in *-607* or *-656* alleles *IL-18 gene* promoter.

**CONCLUSION:**

These data confirm the impact of high serum IL-18 and the haplotype of the polymorphism located in the promoter region of the *IL-18 gene* with the DN.

## Introduction

Diabetic nephropathy (DN) is a chronic complication of both type 1 and 2 diabetes mellitus (DM) [1, 2]. The complications of this disease include kidney failure and high risk to macrovascular problems which may lead to death [2]. Not only diabetic kidney diseases such as Kimmelstiel Wilson disease but also, intercapillary glomerulonephritis include the redundant drain of protein into the urine, hypertension, and steadily defective kidney activity [3]. In severe Kimmelstiel-Wilson syndrome, end-stage renal disease, kidney failure, renal dialysis and the kidney transplant became their occurrence of order.

However, the actual molecular process causing the DN is not extensively clear even though many classic processes and pathways have been suggested to have an impact on DN development. Recently new molecular and epigenetic mechanisms showed evidence that inflammation including secreted proinflammatory cytokines and chemokines is related to DN [4]. Several proinflammatory cytokines such as interleukins (IL-6, IL-8 and IL-18) and tumour necrosis factor-α (TNF-α) are raised in patients with DN [5]. The mode of action for increasing these proinflammatory cytokines is still unpredictable. The oxidative stress has a significant role in raising the nuclear factor-kappaB (NF-κB) [6].

Interleukin-18 (IL-18) is a multiple phenotypic cytokines produced by activated monocytes, dendritic cells and glial cells and it shows impact in numerous inflammatory processes [7]. It belongs to the IL-1 superfamily and it works with IL-12 to induce cell-mediated immunity following infection with microbial products like lipopolysaccharide (LPS). The combination of these two cytokines has been shown to inhibit the IL-4 dependent IgE and IgG1 production and enhance IgG2a production in B cells [8].

Serum IL-18 levels have been showed increasing in patients with DN [9]. IL-18 is known to lead the production of other proinflammatory cytokines [10], endothelial apoptosis [11], upregulation of ICAM-1 [12], and hyperhomocysteinemia [13]. Thus, IL-18 might be an important factor not only in the atherosclerosis processing but also, in the development and progression of diabetic nephropathy.

Two polymorphisms in the *IL-18* gene promoter region at positions *-607* and *-137*, seem to correlate effectively on the genotype and serum concentrations of IL-18 [14]. Hernesniemi et al. (2008) also reported that *IL-18* promoter *G-137C* polymorphism is an important predictor of sudden cardiac death in patients with and without underlying coronary heart disease. In spite of the link between IL-18 promoter polymorphism, diabetes and cardiovascular disease, the relation between an IL-18 promoter polymorphism and cardiovascular disease has not been studied in diabetic nephropathy patients [15].

Position of *IL-18* gene is at chromosome 4q13–21 and several polymorphisms in its promoter region have been identified as *– 607(C/A)* (dbSNP: rs1946518), *-137(G/C)* (dbSNP: rs187238) and - *−656(G/T)* [16]. These common polymorphisms have been shown to regulate the IL-18 production of monocytes and are associated with transcriptional activity of the *IL-18* gene.

This study aimed to determine the association of serum IL-18 level and its gene promoter polymorphism *-607(C/A), -137(G/C)* and *-656(G/T)* with diabetic nephropathy in the Saudi Arabia population as a step towards finding a reliable biomarker for diagnosis the DN disease.

## Material and Methods

### Subjects

Fifty-two patients (40 male and 12 females) diagnosed with Diabetic nephropathy were selected from outpatient Clinic of General AL-Dawadmie Hospital KSA. The diagnosis of DN was done by microalbuminuria (30-300 mg/day) or macroalbuminuria (>300 mg/day) with or without a decrease in glomerular filtration rate (GFR) or arterial hypertension as described by Eknoyan et al., (2003) [17]. Sixty-two diabetic patients without evidence of renal affection were selected as a control group. Both groups were undergone a complete physical and clinical examinations and fasting blood samples were collected. This study was approved by the Local Medical Ethical Committee and according to their instructions. All patients included in the study gave written informed consent. Glycosylated haemoglobin (HbA_1c_), total cholesterol, high-density lipoprotein (HDL), low-density lipoprotein (LDL) and triglyceride (TG) were determined by standard biochemical methods.

### Serum IL-18 assay

The IL-18 was determined in serum samples for each subject by enzyme-linked immune assay (ELISA) (MyBioSource, Cat. No. MBS396299 San Diego, California, USA) according to the manufacture’s instruction.

### DNA extraction

Genomic DNA was purified from whole blood samples with the QIAamp^®^ DNA Blood Mini Kit, Holliston, MA, USA). DNA was eluted in 150 μl elution buffer and examined on 1% agarose gel and stored at –20°C for analysis.

### SNP genotyping and haplotypes reconstruction

PCR-RFLP based method was used to detect polymorphism in an IL-18 gene, for each polymorphism, a specific PCR-RFLP was done. SNPs for, rs1946519 (*−656G/T*) and rs187238 (*−137G/C*) were done by using the method described previously by Folwaczny et al. (2005) [18] while the *IL-18 -607* polymorphism (*C/A*) was identified as described by Kumar et al. (2014) [19]. The primers used for detection are listed in (Table 1) and were ordered commercially from Sigma-Aldrich. PCR reactions were carried on a thermocycler (Bio-Rad, USA) using 2X master mix (Qiagen, Cat No. 206143 Valencia, USA) according to the manufacturer’s instructions. The PCR amplified products were run on 1.5% agarose gel and the bands corresponding to the predicted size were cut and purified using the gel extraction kit (QIA quick columns, Qiagen, Cat No. 28104, Valencia, USA) following the manufacturer’s instructions.

**Table 1:** The primers used in polymerase chain reactions (PCR) for amplification the target region from IL-18 gene

SNPs	PCR primers	Annealing temperature (°C)	Restriction enzymes	Fragment sizes (bp)
*−656 (G/T)*	F:5’AGGTCAGTCTTTGCTATCATTCCAGG’3 R:5’CTGCAACAGAAAGTAAGCTTGCGGAGAGG’3	60	Mwo I	G: 96 + 24 T: 120

*−137 (G/C)*	F:5’CACAGAGCCCCAACTTTTTACGGCAGAGAA’3 R:5’GACTGCTGTCGGCACTCCTTGG’3	60	Mbo II	G: 116 + 39 C: 155

*-607 (C/A)*	F:5’TTCTGTTGCAGAAAGTGTAAAAATTTT’3 R: 5’AAAGGATAGTTGATACAGGCCATT’3	55	Dra I	C: 154 A: 125 +28

### Anthropometry assessment

Anthropometric evaluation was performed for all patients in both groups. Body weight, height and waist circumference were measured following the recommendations of the International Biological Program [20].

Body weight was determined to the nearest 0.01 kg using a Seca Scale Balance, with minimal wear and without shoes. Body height was measured to the nearest 0.1 cm using a Holtain portable Anthropometer. Waist circumference was measured at the level of the umbilicus with the standing position, the face directed forward, shoulders relaxed, and normal breathing by using non-stretchable plastic tape to the nearest 0.1 cm. Body mass index (BMI) was calculated as body weight divided by height squared (kg/m^2^).

### Statistical analysis

Allele’s frequency, genotypes, Linkage disequilibrium and haplotypes were computed using the Arlequin software (version 3.1) and SNPstats (http://bioinfo.iconcologia.net/SN Pstats). Data was presented by means ± SD and percentages. The compiled data were computerised and analysed by SPSS V 12. The following tests of significance were used: t-test between means we used analyses mean difference, t-test between percentage to analyse percent difference and chi – square. A level of significance with p > 0.005 was considered insignificant.

## Results

**Figure 1:**
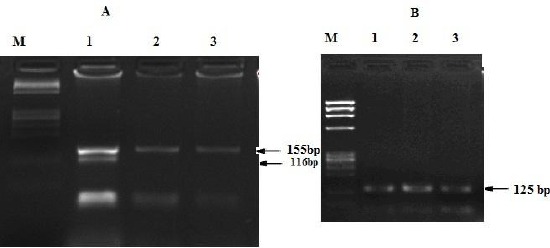
PCR digestion for -137 polymorphism and -607 polymorphism interleukin 18 genes. (A) line 1, -137 GC and lines 2 and 3 -137 CC. M line: ϕx 174 Marker. (B) lines 1,2 and 3 – 607 TT. M line: ϕx 174 Marker

**Figure 2:**
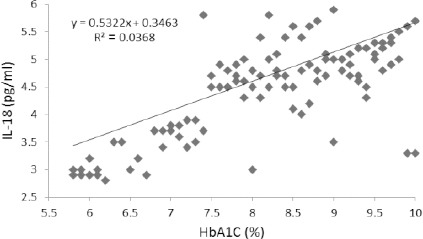
The relation between serum IL-18 and HbA_1c_

In the present work, we analysed serum *IL-18* and three functional polymorphisms, *-137G/C*, *-607C/A* and *-656G/T* at the promoter region of the IL18 gene in 62 DM and 52 DN patients Figure 1. General and clinical characteristics of all the subjects enrolled in this study are shown in Table 2. High serum IL-18 in DN patients in comparison to DM patients is observed as shown in (Table 2). There was significant positive co-relation between serum IL-18 and HBA_1c_ (R^2^ = 0.04, Figure 2).

**Table 2:** General and laboratory characteristics of diabetes mellitus (DM) and diabetic nephropathy (DN) patients

Variable	DM (mean ± SD)	DN (mean ± SD)	P –value
Age (years)	52.8 ± 7.42	52.9 ± 9.15	0.5
Sex (M/F)	47/15	40/12	--
BMI (kg/m^2^)	24.1 ± 5.1	28.3 ± 5.2	0.05*
SBP (mmHg)	133.6 ± 20.1	147.6 ± 27.9	0.005*
DBP (mmHg)	80.1 ± 15.5	99.0 ± 15.2	0.005*
Smoking habit %	40.3%	63.5%	0.005*
S. cholesterol (mmol/l)	3.6 ± 1.1	3.8 ± 1.3	0.2
S. LDL (mmol/l)	2.3 ± 0.9	2.5 ± 1.1	0.2
S. HDL (mmol/l)	1 ± 0.4	1.1 ± 0.4	0.1
S. triglyceride (mmol/l)	1.5 ± 0.5	1.7 ± 0.6	0.1
S. urea (mg/dl)	33.6 ± 12.3	47.3 ± 20.7	0.05*
S. creatinine (µmol/l)	77.3 ± 21.1	130.5 ± 27.6	0.04*
Cr Cl (mL/min)	122.9 ± 15.3	68.8 ± 9.9	0.005*
UACR (mg/mmol/L)	25.4 ± 10.1	53.6 ± 13.3	0.04*
IL-18 (pg/ml)	3.1 ± 0.4	5.2 ± 1.5	0.01*
HbA_1c_	8.4 ± 2.5	8.9 ± 2.1	0.4
Oral drug therapy	57 (91.9%)	39 (75%)	--
Insulin drug therapy	5 (8.1%)	13 (25%)	--
Albuminuria (mg/24h)	24.5 ± 5.2	373.7 ± 44.2	0.001*

Cr Cl (Creatinine clearance) = 1.23 x (140-age in years) x weight (kg) / s. creatinine (µmol/l);

* Significance between DM and DN patients (P < 0.05).

Comparing the allele frequencies of *-137G/C*, *607C/A* and *656G/T* at the *IL-18* promoters polymorphisms among the DM and DN patients was indicated in Table 3 and Table 4 revealed significant ratio of *-137G* allele (about 80.8%) more than *C* allele in DN (P < 0.05) and in DN than DM patients (P < 0.05) while no significant variation between both groups in *-607C/A* and *–656G/T* polymorphism was observed.

**Table 3:** Comparing alleles of IL-18 promoter polymorphisms in DM and DN

*Alleles*	DM (%)	DN (%)
*-656* *G* *T*	(51.6%) (48.4%)	(48.1%) (51.9%)
*-607* *C* *A*	(46.8%) (53.2%)	(50%) (50%)
*-137* *C* *G*	(53.2%) (46.8%)*	(19.2%)** (80.8%)

* : Significance between DM and DN in *-137 G* allele (P < 0.05);

** : Significance between *C* and *G* alleles of -137 in DN patients (P < 0.05).

**Table 4:** *IL-18* Haplotypes distribution in diabetes mellitus (DM) and diabetic nephropathy (DN)

DN N (%)	DM N (%)	Haplotypes
*-137C/-607A/-656G*	9 (14.5 %)	3 (5.8 %)*
*-137C/-607A/-656T*	10 (16.1 %)	3 (5.8 %)*
*-137C/-607C/-656G*	7 (11.3 %)	2 (3.8 %)*
*-137C/-607C/-656T*	7 (11.3 %)	2 (3.8 %)*
*-137G/-607A/-656G*	7 (11.3 %)	10 (19.2%)
*-137G/-607A/-656T*	7 (11.3 %)	10 (19.2 %)
*-137G/-607C/-656G*	7 (11.3 %)	12 (23.1)
*-137G/-607C/-656T*	8 (12.9 %)	10 (9.2%)

* : Significance between DM and DN in the same haplotype (P < 0.05).

## Discussion

The patients with diabetic nephropathy, in general, suffer from interruption of angiogenesis, permeability, apoptosis, and multiplication which could be as a result of inflammatory processes which affected the immune cells and cause the fibrotic phenomenon. This disease is characterised by the evolution from normoalbuminuria to microalbuminuria followed by continuous diminish in the glomerular filtration rate (GFR), and high arterial blood pressure.

IL-18 is a dominant inflammatory cytokine that induces IFN-γ [21] which in turn induces functional chemokine receptor expression in human mesangial cells [22].

In addition, IL-18 leads to the production of IL-1, and TNF−γ and upregulation of ICAM-1, as well as apoptosis of endothelial cells [23]. IL-18 is constitutively expressed in renal tubular epithelia [17], infiltrating monocytes, macrophages, and T cells, endothelial cells of interstitial vessels along with proximal renal tubular cells, are potential sources of this cytokine [24]. In this study, *IL-18* promoter polymorphism *-137* is associated with the development of nephropathy in diabetic patients.

The distribution of genotypes in the current study is similar to previous reports on Chinese subjects. For example, we found that the *-137G* allele (*CG* or *GG* genotypes) was more common in DN than *C* allele (*CG* or *CC* genotypes) which is similar to Dong et al., 2007 and Szeto et al., 2009. Dong et al., 2007 found that *GG, GC* and *CC* genotypes at the *-137* site were 71.8%, 25.0% and 3.2% respectively but 78.7%, 20.0% and 1.3% respectively as reported by Szeto et al., 2009 [25, 26].

Further, among white males, the *C* allele carriers at the *-137* position had a high mortality risk but that with normal renal function and associated with high risk of cardiovascular disease [15]. A change at *IL-18* gene promoter at *-137* from *G* to *C* can change the histone 4 transcription factor-1 (H4TF-1) nuclear factor binding site to a binding site for an unknown factor found in the granulocyte-monocyte colony stimulating factor (GM-CSF) promoter [19]. Similarly, we found that the *-607(C/A)* and -656(G/T) had no significant difference in DM and DN which is similar to data reported by Dong et al., 2007 and Szeto et al., 2009 [25, 26]. Although other factors may also affect *IL-18* gene expression, available data suggest that promoter polymorphism is the major determinant of IL-18 production. Hyperglycemia stimulates the synthesis of IL-18 [27].

In the current study, it was found a statistically significant relationship between serum of IL-8 and the levels of glycosylated haemoglobin, in patients with diabetic nephropathy. Not only hyperglycemia but also albuminuria stimulates *IL-18* expression in proximal tubular cells [28] which is correlated with our results.

The authors are very grateful to patients and their family for their participation and cooperation during this study. We did not have funding body for this study, all was funded by authors. However, the authors are thankful to Shaqra University, Ministry of Higher Education, Kingdom of Saudi Arabia for laboratory facility to do this research. We are also very much thankful to the Department of Clinical Biochemistry Laboratory, Faculty of Medicine AlDawadmi, Shaqra University Kingdom of Saudi Arabia for their help with lab facility. We may not proceed to this present work if they do not allow us. We again thank their ethical committee for approving the work.

**Abbreviations**
DM: diabetes mellitus
DN: diabetic nephropathy
GFR: glomerular filtration rate, IL-18: Interleukin- 18
NKC: natural killer cell, IFN-γ: interferon-γ
ICAM-1: Intercellular Adhesion Molecule 1
